# The link between BDNF and platelets in neurological disorders

**DOI:** 10.1016/j.heliyon.2024.e39278

**Published:** 2024-10-11

**Authors:** Nezha Bouhaddou, Meryem Mabrouk, Farah Atifi, Abdelhakim Bouyahya, Younes Zaid

**Affiliations:** a Physiology and Physiopathology Team, Genomics of Human Pathologies Research Center, Faculty of Sciences, Mohammed V University, Rabat, Morocco; b Laboratory of Materials, Nanotechnology and Environment, Faculty of Sciences, Mohammed V University, Rabat, Morocco; c Laboratory of Human Pathologies Biology, Department of Biology, Faculty of Sciences, Mohammed V University, Rabat, Morocco; d Immunology and Biodiversity Laboratory, Department of Biology, Faculty of Sciences, Hassan II University, Casablanca, Morocco

**Keywords:** BDNF, Platelets, Neurological disorders

## Abstract

Platelets are considered one of the most important reservoirs not only of growth factors, but also of neurotrophic factors that could contribute to the repair of vascular lesions and the prevention of neurological deterioration. Among these factors, Brain-Derived Neurotrophic Factor (BDNF) - a protein belonging to the neurotrophin family - is widely expressed both in the hippocampus and in platelets. Platelets constitute an important reservoir of BDNF; however, little is known about the factors modulating its release into the circulation and whether anti-platelet drugs affect this secretion. In this review, we have discussed the link between BDNF and platelets and their role in neurological disorders.

## 1 Introduction

BDNF belongs to the neurotrophin family (NTs) and is primarily known for its role in the differentiation and morphology of neurons, synaptic plasticity, and memory in the central nervous system (CNS) [[1], [2], [3], [4], [5]]. The neuronal function of BDNF is primarily mediated by its receptor tyrosine kinase, TrKB, found on synaptic terminals. Two forms of extracellular BDNF are present: pro-BDNF (the precursor form of the mature protein) and mature BDNF, released into the extracellular space and found in circulation [6,7] (see Table 1).

**Table 1:** The interplay between BDNF and Platelets in neurological disorders.

**Neurological disorder**	**BDNF involvement**	**Platelets' implication**	**Mechanisms under Investigation**	**Signaling pathways disturbed**	**Clinical relevance**	**References**
Alzheimer's Disease	Reduced BDNF levels in critical brain regions, such as the hippocampus and cortex	Platelets as BDNF reservoir	BDNF transport and release	Hippocampal plasticity, synaptic plasticity, memory formation & neuronal survival	Potential therapeutic approaches targeting BDNF modulation	[109,129]
Parkinson's Disease	BDNF localization in dopaminergic neurons & Platelets-derived BDNF role in neuroprotection	BDNF release by platelets and role in PD progression	Platelet-derived BDNF role in dopaminergic neurons protection	Dopaminergic signaling pathways & neuronal survival	Therapeutic interventions in Parkinson's Disease	[112]
Multiple Sclerosis (MS)	BDNF involvement in disease progression.	N/A	Role of BDNF in MS	Inflammatory pathways, immune response	Therapeutic approaches and a better understanding of disease progression	[130,131]
Depression	Reduced BDNF levels in the hippocampus and cortex	Potential role in mood regulation	BDNF transport and release & inflammatory factors	Synaptic plasticity & mood regulation	Novel therapeutic approaches targeting BDNF modulation	[132,133]
Schizophrenia	BDNF involvement in neurodevelopment	Potential developmental presence of BDNF in platelets	Role of platelets in schizophrenia	Neurodevelopmental pathways, synaptic plasticity, mood regulation	Potential therapeutic approaches targeting BDNF modulation	[[134], [135], [136]]
Autism Spectrum Disorder (ASD)	BDNF involvement in neural development	Potential developmental presence of BDNF in platelets	BDNF's involvement in neural development, as well as the specific role of platelet derived BDNF in ASD.	Neurodevelopmental pathways, synaptic plasticity	Potential therapies and interventions to address neurodevelopmental aspects	[11,78,137]
Neuropathic Pain	Altered BDNF levels in the brain	Altered BDNF levels & platelets' potential role in pain signaling	BDNF in neuropathic pain	Pain-related signaling pathways	Novel treatment strategies development	[124,138,139]
Traumatic Brain Injury (TBI)	Altered BDNF levels in the brain.	N/A	BDNF and platelets contribution to neurorepair	Neurorepair signaling pathways, neuronal survival	Potential therapies that aid in neurorepair and neuronal survival	[9,90,140]
Stroke	BDNF involvement in stroke recovery		BDNF's role in neurorepair	Neurorepair signaling pathways, neuronal survival, neuroprotection		[141,142]
Spinal Cord Injury (SCI)	BDNF involvement in neurorepair.	Potential platelet involvement in SCI	Neuroregeneration			[143,144]

In addition to its central role in the nervous system, recent research has highlighted an important peripheral component of BDNF activity: its presence and function in platelets [8,9]. Platelets, traditionally known for their role in thrombosis and haemostasis, have been shown to be a major peripheral reservoir for BDNF [10,11]. This dual role of platelets, both in hemostasis and as carriers of BDNF, suggests a complex interplay between hematological and neurological health [9,[11], [12], [13], [14], [15]]. Understanding this relationship opens up new avenues for studying how BDNF stored in platelets may impact on neurological disorders and potential treatment strategies.

The aim of this study is to analyze the complex relationship between BDNF and platelets, and their implications in neurological disorders. We will study the process of BDNF storage and release by platelets, the elements that influence its release, and the potential influence of antiplatelet drugs on this process. By focusing on the role of platelets as reservoirs of BDNF, we aim to highlight possible therapeutic strategies for different neurological disorders where BDNF dysregulation is evident.

## 2 BDNF and platelets: biological mechanisms

### 2.1 Brain-derived neurotrophic factor structure, expression, and localization of BDNF

Brain-Derived Neurotrophic Factor (BDNF), a member of the neurotrophin family, is encoded by the BDNF gene, located on human chromosome 11p14.1 [16]. It is synthesized as a precursor called proBDNF, which is later cleaved to form the mature, biologically active BDNF protein consisting of 119 amino acids [17]. This mature BDNF protein exists as a stable homodimer and is known for its significant role in neural survival and differentiation [18].

BDNF is widely distributed throughout the nervous system, with notably high expression in regions critical for cognitive processes, including the hippocampus and the cortex [[19], [20], [21]]. It can be found in various neuronal cell types, including pyramidal neurons, interneurons, and glial cells.

BDNF's predominant localization within synaptic terminals is crucial for activity-dependent synaptic plasticity [20,22,23]. It is stored in vesicles and released upon neuronal activation, where it functions as a signaling molecule [24]. BDNF expression is regulated by various factors, including neuronal activity, synaptic transmission, and neurotrophic signals [25].

BDNF is also expressed in various peripheral tissues and diverse cell types, including immune cells and skeletal muscle [26]. It has notably been detected in platelets, which are recognized for their roles in blood clotting, inflammation, and immune responses [9]. The discovery of BDNF in platelets suggests potential peripheral functions and intriguing connections to neurological processes [27]. The extensive distribution of BDNF underscores its roles, not only within neural processes but also in non-neural functions.

### 2.2 BDNF functions, receptors, and signaling pathways

BDNF exerts different functions such as neuroprotection, neuronal survival, and neuronal protection against oxidative stress and excitotoxicity through intricate interactions with its receptors and intracellular signaling pathways [28]. It plays a pivotal role in neuronal differentiation, promoting the formation of neural circuits by supporting axonal and dendritic growth, synaptogenesis, and refining neural connections [28]. BDNF serves as a linchpin in synaptic plasticity, regulating long-term potentiation (LTP) and long-term depression (LTD) while shaping the strength of synaptic transmission, thus contributing to learning and memory [29]. Furthermore, BDNF impacts various neurotransmitters release, including glutamate, GABA, and dopamine, affecting synaptic transmission and network activity [[30], [31], [32], [33]]. Its involvement in regulating the hypothalamic-pituitary-adrenal (HPA) axis and maintaining energy homeostasis renders BDNF relevant to stress responses and metabolic disorders [[34], [35], [36], [37]].

BDNF exerts its functions through intricate interactions with specific receptors. The high-affinity receptor, TrkB (Tropomyosin receptor kinase B), stands as the principal molecular gateway for BDNF [28,38]. TrkB, which plays a pivotal role in mediating BDNF's neurotrophic actions, is ubiquitously distributed across neurons in the central nervous system [39]. When BDNF binds to TrkB, it triggers a cascade of intracellular events, involving key signaling pathways such as PI3K/Akt, MAPK/ERK, and others, which collectively lead to an array of neurotrophic effects [[39], [40], [41]].

TrkB receptors are not a monolithic entity; instead, they exist in multiple isoforms, adding a level of complexity to BDNF signaling. These isoforms, including full-length TrkB (TrkB-F) and truncated variants like TrkB-T1, modulate BDNF's actions in specific cellular contexts, thereby influencing the multifaceted outcomes of BDNF signaling [[39], [40], [41], [42]].

In addition to TrkB, p75NTR (p75 Neurotrophin Receptor), a low-affinity receptor for various neurotrophins, plays a modulatory role in BDNF signaling [43]. It collaborates with TrkB to amplify BDNF's signaling effects, enhancing the affinity of TrkB for BDNF and significantly influencing downstream outcomes [44,45]. p75NTR contributes to the complexity and variability of BDNF-mediated functions through its multifaceted modulatory roles.

The receptors, TrkB and p75NTR, serve as crucial mediators of BDNF functions. BDNF's interactions with these receptors underpin the intricate mechanisms that drive neuroprotection, neural differentiation, synaptic plasticity, neurotransmitter regulation, and neuroendocrine homeostasis. Understanding the roles of these receptors provides insights into how BDNF orchestrates its functions and contributes to the maintenance and development of the nervous system.

### 2.3 Platelets: beyond haemostasis

Platelets are small discoid cells, approximately 2–5 μM in diameter, with a circulating lifespan of about 10 days [46]. Their distinctive structure, marked by the lack of a nucleus, and their function in hemostasis, make them unique. The platelet cytoskeleton is formed by a dense network of actin filaments and tubulin microtubules. This network of filaments undergoes significant rearrangement during platelet activation, enabling platelets to change shape from a discoid to a flattened form with pseudopodia [46,47]. Platelets contain several organelles, including mitochondria, lysosomes, and various granules. During activation, platelets release a multitude of small molecules, proteins, and growth factors that are usually stored in their granules, namely dense granules, and α-granules. These granules are pre-formed in megakaryocytes and continue their maturation in platelets [[48], [49], [50]]. The largest and most abundant are the α-granules, which contain adhesion proteins, various membrane receptors (Glycoprotein VI -GPVI- and αIIbβIII), fibrinogen, fibronectin, von Willebrand factor, and growth factors such as platelet-derived growth factor (PDGF), vascular endothelial growth factor (VEGF), and brain-derived neurotrophic factor (BDNF) [14,51]. Dense granules contain small soluble molecules often released rapidly, such as ADP, serotonin, cations like Ca^2+^, Mg^2+^, and part of platelet P-selectin [52,53]. Thanks to the high proteomic diversity of platelets, they play a primary role in haemostasis. When in contact with a damaged vessel, platelets become activated, initiating a positive feedback loop for recruiting other platelets to the injury site, releasing crucial coagulation factors, and sealing the vascular breach [54].

Platelets are also involved in various other physiological phenomena [55,56], contributing to angiogenesis, inflammation [57], and the immune response. It is increasingly evident that they also play a role in neurological diseases. Platelets contain numerous proteins involved in the regulation of angiogenesis, stored in α-granules [58,59]. and contain both pro- and anti-angiogenic factors, released differentially depending on the stimulus for platelet activation [[60], [61], [62]], though this remains controversial with opposing study results [[63], [64], [65]]. Functionally, platelet release as a whole has a global effect favoring angiogenesis [65]. The angiogenic effect of platelet release is highly relevant at the biological level. Since platelet activation occurs at sites of vascular injury, promoting the division and migration of endothelial cells, including the release of vascular endothelial growth factor (VEGF), ultimately involved in the repair of vascular damage [66,67]. However, these same angiogenic properties may also be involved in pathological processes, such as platelets promoting angiogenesis for tumor survival and growth [59].

Platelets are also involved in the inflammatory and immune response. In addition to angiogenic proteins, α-granules contain numerous cytokines [58,68]. These include chemokines and interleukins regulating the immune response [58,68,69], such as platelet factor 4 (PF4), recruiting monocytes and differentiating them into macrophages [70,71]. Some proteins secreted by platelets also have a direct antimicrobial effect on certain types of microorganisms [72].

For a long time, platelets have been considered a peripheral and easily accessible model of neurons due to the many similarities between these cell types [[73], [74], [75], [76]]. Initially used for studying serotonin transport and the impact of antidepressant drugs [74,77], platelets are now explored as a model for discovering biomarkers for neurological and neurodegenerative diseases [[78], [79], [80]].

Neurotransmitters are an integral part of neuronal communication and are essential for the propagation of nerve impulses. Like neurons, platelets are capable of internalizing, storing, and secreting neurotransmitters, including dopamine, adrenaline, GABA, and serotonin [74,[81], [82], [83]]. Of these, serotonin is best characterized in platelets. It has been reported in the literature that over 99 % of the serotonin found in circulation is contained in platelets [76]. Platelets acquire serotonin by internalization from the bloodstream via the serotonin transporter SERT [84]. Once inside the platelet, serotonin can be transported into the dense granules via the vesicular monoamine transporter 2 (VMAT2) found on their membrane, or degraded by the monoamine oxidases present in platelets [78,85]. Once released from the dense granules, serotonin contributes to platelet activation and vasoconstriction, thereby aiding in the cessation of bleeding [86].

Platelets also contain proteins known for their roles in the brain [76]. Among them are several isoforms of the amyloid protein precursor (APP), whose beta-amyloid metabolite appears to be involved in the pathophysiology of Alzheimer's disease. The ratio of different APP isoforms found in platelets has also been associated with Alzheimer's disease, and this ratio has been proposed as a potential peripheral biomarker for this neurodegenerative disease [87].

### 2.4 BDNF and platelets in neurological disorders: comparison of brain and serum levels

BDNF dysregulation has emerged as a key factor in several neurological and psychiatric disorders, offering valuable insights into the potential therapeutic dimensions of these conditions [88,89]. Reduced BDNF levels have been consistently observed in a spectrum of disorders, including Alzheimer's disease, Parkinson's disease, depression, and schizophrenia [28]. These neurological disorders are associated with alterations in BBB function [76]. These changes can be caused by inflammatory processes, oxidative stress or endothelial dysfunction, leading to increased BBB permeability [76]. As a result of this increased permeability, circulating BDNF is able to cross the BBB more easily and gain access to the brain parenchyma [76].

The collaboration between BDNF, platelets, and psychiatric disorders presents a promising avenue for understanding and potentially treating these complex mental health conditions [90].

The existing association between circulating and cerebral levels of BDNF in certain experimental studies has given rise to the idea that blood levels of BDNF are a reliable reflection of the levels of BDNF present in the brain [91]. This aspect requires confirmation since, in humans, BDNF is assessed in the bloodstream.

BDNF released by human blood platelets is the main factor contributing to BDNF levels in serum [92]. Since mice do not have BDNF in their platelets, this confounding factor is not present in them. BDNF levels in mouse serum and plasma were approximately one thousand times lower than those observed in human serum [92]. This study also points out that the main source of BDNF in rodent blood is skeletal muscle, not platelets [92].

Although the presence of BDNF in platelets is well established, its origin and mechanisms of action remain partially understood. It has been suggested that BDNF may be internalized by platelets from the circulation, or inherited from megakaryocytes, its precursor cells [9].

Several studies have demonstrated that BDNF is secreted during platelet activation [9,13,16,93]. The first evidence for the platelet origin of circulating BDNF was the observation of much higher levels of BDNF in serum (up to 100–200 times) than in plasma (1–10 ng/mL), suggesting that coagulation released large quantities of the neurotrophin into the circulation [16]. The pathways involved in BDNF release from platelets were further investigated in purified systems, where many classical agonists were able to induce the secretion of appreciable levels of BDNF from intraplatelet stores in their milieu [9,93,94]. In 2001, Tamura et al. [94] identified two different pools of BDNF contained in human platelets, one found in the α-granules and the other in the cytoplasm. As a result, a large proportion of platelet BDNF is released during activation, while the remainder remains within the platelet [93,94].

In a study of 1385 participants, roughly equally divided between those with and without Coronary Artery Disease (CAD), Bélanger et al. [95] investigated whether BDNF levels mediated the relationship between platelet activation and cognitive function, and whether this relationship was moderated by the presence of CAD. While platelet hyperactivity is generally deleterious in coronary artery disease and is associated with poorer cognitive outcomes, platelets also release large amounts of BDNF during activation. Mediation modeling showed that BDNF levels attenuated the association between platelet hyperactivity and cognitive decline, with BDNF having a weaker protective effect on cognitive function in participants with CAD than in controls [95]. This suggests that BDNF released by platelets may have a protective effect against cognitive decline. In support of this hypothesis, Weinstein et al. [96] showed in the original Framingham cohort (5209 participants aged 28–62 with normal cognitive performance at recruitment) that lower serum BDNF levels preceded the onset of cognitive impairment over a 10-year follow-up period. As serum BDNF levels reflect platelet BDNF stores [13,93], it is possible that increasing circulating BDNF levels may lead to better cognitive outcomes.

BDNF dynamics are complex, and accurate measurement techniques are essential, as a recent study highlights [97]. It also highlights the interest of BDNF as a biomarker for various neurological conditions, and the importance of taking into account disparities between species in research [97].

## 3 BDNF and depression

In individuals with depression, reduced BDNF levels, particularly in critical brain regions such as the hippocampus and prefrontal cortex [98,99], correlate with the atrophy observed in these regions, a hallmark of depression [100]. Various treatments for depression, such as selective serotonin reuptake inhibitors (SSRIs) and electroconvulsive therapy (ECT), have been shown to increase BDNF levels [[101], [102], [103], [104]].

Studies respectively comparing serum and plasma BDNF levels in depressed and healthy subjects found lower levels of BDNF in depressed subjects, with more reproducible results between studies for serum than plasma BDNF [101,105]. Additionally, antidepressant treatments seem to increase serum BDNF in depressed subjects [106,107]. However, the existence of an association between depression and low circulating levels of BDNF does not necessarily imply that the cause of the reduction in circulating BDNF is a reduction in brain levels. A recent study in a genetic model of depression even showed an inverse correlation between circulating and brain BDNF [108].

## 4 BDNF and Alzheimer's disease

In Alzheimer's disease, reduced BDNF levels contribute significantly to cognitive decline [109]. Restoring BDNF levels or enhancing its signaling pathways are potential therapeutic strategies [109,110].

Serum levels of BDNF are lower in patients with Alzheimer's disease than in control subjects [111] and increase when treated with an acetylcholinesterase inhibitor [132]. Similar results were found in patients with mild cognitive impairment without dementia [133]. However, these findings remain controversial, as other studies have shown opposite results, suggesting an increase in serum BDNF in the mild stages of the disease compared to levels observed in more advanced stages and in healthy subjects [[134], [135], [136]]. Finally, Alzheimer's patients with the val66met polymorphism have a 2 to 3-fold increased risk of depression [137].

## 5 BDNF and Parkinson's disease

Similarly, in Parkinson's disease, characterized by the progressive loss of dopaminergic neurons, BDNF has emerged as a promising factor for enhancing neuronal survival and function [112]. Ongoing clinical trials are exploring BDNF-based therapies for Parkinson's disease [112,113].

Recent studies revealed significantly lower serum BDNF levels in patients, with a positive correlation between levels and the duration and severity of the disease [111,114].

**Fig. 1:**
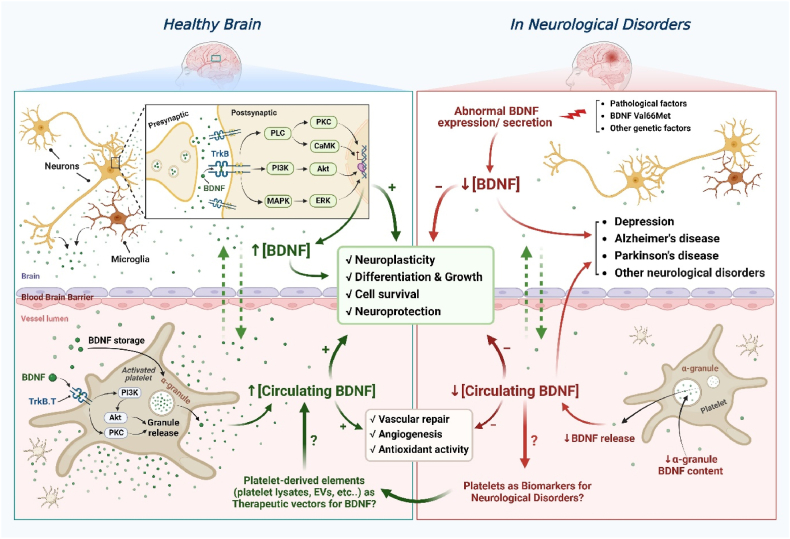
Summary of the role of BDNF and its link with platelets in neuronal and vascular health and disease. The interaction between BDNF and TrkB in neuronal cells induces the activation of three intracellular signaling pathways (PLC, PI3K, and MAPK), thus promoting the development and survival of neurons. The binding of BDNF to TrkB.T on platelets mediates the release of granular content, including BDNF, via the PI3K and PKC pathways, leading to increased levels of circulating BDNF, which contribute to vascular repair [11]. BDNF can potentially cross the blood-brain barrier between the bloodstream and brain in both directions, maintaining high levels in both compartments, thus improving brain and vascular health [153]. Under abnormal conditions, pathological and genetic factors can influence the expression and/or secretion of BNDF stored in neuronal cells and α-granules of platelets, leading to its decreased levels in circulation and the brain, potentially promoting the development of different neurological disorders (depression, Alzheimer's disease, Parkinson's disease, etc.) [129,[153], [154], [155], [156], [157], [158]]. Platelet-derived elements, including EVs and platelet lysates, serving as reservoirs of BDNF, could potentially be used as therapeutic vectors to increase BDNF levels in circulation, potentially enhancing brain and vascular health [90]. Akt: protein kinase B; BDNF: Brain-Derived Neurotrophic Factor; CaMK: calcium/calmodulin-dependent protein kinase; ERK: extracellular signal-regulated kinase; EVs: extracellular vesicles; MAPK: Mitogen-Activated Protein Kinases; PI3K: phosphoinositide 3-kinase; PKC: protein kinase C; PLC: phospholipase C; TrkB: tropomyosin receptor kinase B; TrkB.T: truncated form of TrkB; α-granule: alpha granule.

Fig. 1 summarizes the role of BDNF and its link with platelets in neuronal and vascular health and disease.

## 6 BDNF and stroke

Circulating levels of BDNF in stroke are poorly documented, and the available data have all been obtained in humans. The first study on this subject involved 10 patients with a first cerebral infarction less than 24 h old [115]. BDNF was measured in plasma using an ELISA technique on admission and then repeatedly in the morning at 8 a.m. over the following 4 days. Despite a break in the blood-brain barrier, as evidenced by the increase in plasma S100β protein levels measured concomitantly, the authors reported stable plasma BDNF levels. However, interpretation of the results of this study remains difficult given the small number of patients included, few of whom had severe neurological deficits, the delay in the first sampling, which varied greatly from one patient to another (10.7 h ± 7.6), and the absence of data on the final volume of the lesion. The other three studies looked at the BDNF-depression pair in stroke patients. In a study including 134 patients with cerebral infarction, Jiménez et al. (2009) [116] measured serum BDNF at 7 ± 2 days and 30 ± 7 days using an ELISA technique. No difference was found between patients who developed depression in the month following the infarction (22.1 % of all patients) and those who did not. More recently, Yang et al. (2011) studied 100 patients admitted within 24 h of a first cerebral infarction [117]. Serum BDNF was measured the morning after admission and on the seventh day using ELISA technique. Three groups of subjects were selected: patients with depression diagnosed within 14 days of cerebral infarction, patients without depression, and control subjects. The authors report higher serum BDNF levels at Day 1 in the group of patients without depression compared with the group of patients with depression and control subjects, with no significant difference observed at Day 7. After multivariate analysis, including admission clinical and radiological data, a low serum BDNF level at Day 1 was independently associated with an increased risk of depression in patients with cerebral infarction. A level of less than 5.86 ng/ml predicted this risk with a sensitivity of 81 % and a specificity of 86 %. The authors concluded by reverse reasoning that higher serum BDNF levels could reflect brain BDNF levels, which are known to have a beneficial effect on cerebral plasticity, and thus provide protection against the onset of post-stroke depression. Similarly, in another study of 93 patients with cerebral infarction, Zhou et al. (2011) [118] found that serum BDNF levels measured between 3 and 6 months were significantly lower in patients who developed post-stroke depression.

## 7 BDNF and other neurodegenerative diseases

### 7.1 BDNF in amyotrophic lateral sclerosis (ALS) and Huntington's disease

ALS and Huntington's disease, involve BDNF, which contributes to motor neuron degeneration and striatal atrophy [89,119].

### 7.2 BDNF in schizophrenia

schizophrenia, a complex neuropsychiatric disorder characterized by cognitive deficits and adverse symptoms, has been another focus of interest in the context of BDNF. Reduced BDNF levels have been observed in individuals with schizophrenia, contributing to the intricate neurobiological landscape of the disorder [120,121]. While the precise mechanisms are complex and still under investigation, it is evident that BDNF plays a role in the neurodevelopmental aspects of schizophrenia. BDNF's involvement in schizophrenia extends beyond mere reduction in levels; it is intertwined with the complex interactions within the brain regions involved in the disorder [120,122].

### 7.3 BDNF in peripheral neuropathy

Peripheral neuropathy is linked to low levels of BDNF [28,123], highlighting its role in maintaining the health of sensory neurons in the peripheral nervous system. The aim of strategies to improve BDNF signaling is to reduce symptoms and promote nerve regeneration [124]. BDNF is also a critical player in supporting sensory neurons affected by diabetic neuropathy. Dysregulation of BDNF contributes to the development and progression of this condition [125].

### 7.4 BDNF in fibromyalgia and **chronic** regional pain syndrome

Chronic pain disorders, such as fibromyalgia and chronic regional pain syndrome, often have a peripheral component [126]. BDNF is central to pain signaling and sensitization [124]. In this context, dysregulation of BDNF signaling in peripheral tissues is associated with chronic pain conditions' onset and maintenance [127]. The quest to improve the management and treatment of these disorders, driven by the complex role of BDNF, represents a promising perspective in medical research and therapeutic innovation.

### 7.5 BDNF in systemic inflammation

According to a recent study, systemic inflammation leads to a significant decrease in blood levels of BDNF [128]. This decrease is attributed to the influence of inflammatory cytokines on BDNF production and release [128]. The findings of this research have significant clinical implications, particularly for neurological and psychiatric disorders, which could worsen the already low levels of BDNF and further impair neuroplasticity and cognitive function.

Although our study shows a significant correlation between serum BDNF levels and neurological disorders, it should be emphasized that direct mechanistic evidence of the relationship between the two has yet to be established. Further research is essential to understand the precise mechanisms by which platelet-derived BDNF might impact neurological disease. This lack of direct mechanistic evidence is an essential area for future study, paving the way for further investigation into the therapeutic potential of BDNF in these circumstances.

**Fig. 2:**
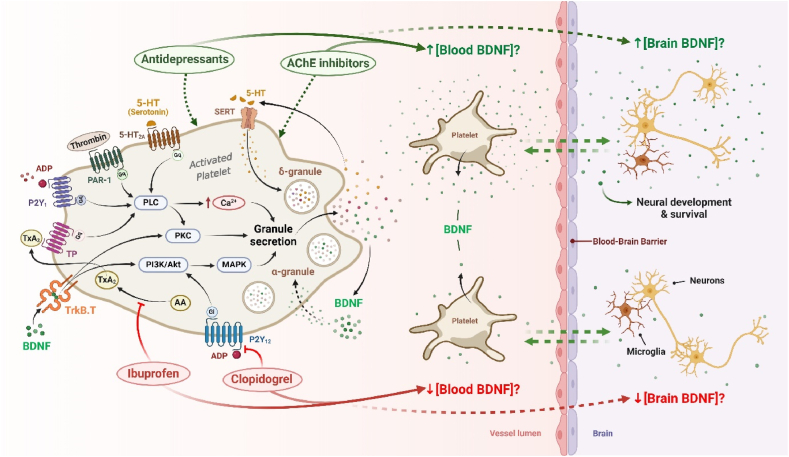
**Platelet BDNF release signaling pathways and potential strategies for their modulation**. Various soluble agonists, such as thrombin, ADP, 5-HT, and TxA2, bind to their respective receptors and promote intraplatelet signal transduction. The activation of Gq-protein-coupled receptors (PAR-1, P2Y1, 5-HT2A, and TP receptors) stimulates PLC, inducing intracellular Ca2+ mobilization and PKC activation. Activation of the Gi-protein-coupled receptor (P2Y12) mediates the PIK3/Akt/MAPK pathway. Both pathways lead to the secretion of granules containing various soluble mediators such as ADP, 5-HT (stored in δ-granules), and BDNF (stored in α-granules) [8,9]. Platelets express a truncated form of TrkB (TrkB) on their surfaces [11]. BDNF-TrkB.T binding induces platelet activation and granule release by stimulating PI3K/Akt/MAPK and PKC pathways [11]. Both antidepressant and AChE inhibitor treatments can modulate platelet BDNF secretion, resulting in an increase in circulating BDNF levels, potentially raising BDNF levels in the brain [159,160]. Ibuprofen and clopidogrel are agents that inhibit the release of granular content, including BDNF, which can lead to a decrease in circulating BDNF levels and potentially brain BDNF levels [145,161]. Note: these potential mechanisms for modulating platelet BNDF release require further investigations. AA: arachidonic acid; AChE: acetylcholinesterase; ADP: adenosine diphosphate; Akt: protein kinase B; BDNF: Brain-Derived Neurotrophic Factor; MAPK: Mitogen-Activated Protein Kinases; PI3K: phosphoinositide 3-kinase; PKC: protein kinase C; PLC: phospholipase C SERT: serotonin transporter; TP: thromboxane receptor; TrkB.T: truncated form of tropomyosin receptor kinase B; TxA2: thromboxane A2; α-granule: alpha granule; δ-granule: dense granule; 5-HT: serotonin; 5-HT_2A_: serotonin 2A receptor.

Fig. 2 illustrates the signaling pathways involved in Platelet BDNF release, along with potential approaches to regulate these pathways.

The following table provides an overview of the interplay between BDNF and platelets in neurological disorders, facilitating further exploration and understanding.

## 8 Potential treatment strategies

### 8.1 Strategies to modulate BDNF and platelet function for therapeutic purposes

Even though platelets constitute the most important reservoir of BDNF and release it in large quantities into the circulation, little is known about the factors that influence its release into the bloodstream. Although the effects of antidepressants on circulating levels of BDNF are increasingly being studied, few studies exist on the effects of antiplatelet drugs on its secretion. A single study published in 2011 investigated the impact of Aspirin and Clopidogrel on BDNF release. Although the study showed that a single oral dose of Clopidogrel, but not Aspirin, significantly reduced platelet BDNF release in healthy volunteers, the links between platelet function in the presence of these drugs and BDNF were not demonstrated [145].

Jones et al. (2008) studied the effect of tachykinins, classical neurotransmitters, on platelet function [146]. These molecules have the same implications as BDNF in inflammation and cardiac contraction, and also act in an autocrine/paracrine manner [147]. Their research shows that platelets store tachykinins and that these are released after platelet activation in the same way as BDNF [146]. Not only are these tachykinins released, but they also bind to their receptors on the platelet surface and induce aggregation dependent on secondary mediators and inhibitable by current anti-platelet therapies (aspirin and clopidogrel). Their results suggest that tachykinin-induced aggregation is significant, since once tachykinins were inhibited, thrombus formation and size were reduced in vitro. In vivo, tachykinins play a role in haemostasis by reducing thrombi formation in mouse models of thrombosis [146].

Conversely, molecules active specifically on platelets and megakaryocytes, such thrombopoïétine (TPO), also have neuronal effects. On the one hand, TPO reduces cerebral ischemic damage and improves post-ischemic cerebral function by inhibiting the metalloproteinase MMP-9 and protecting the blood-brain barrier in animals [148]. On the other hand, it has a proapoptotic effect on new neurons via the Ras rhoGTPase and is inhibited by the presence of neurotrophins. These results suggest that TPO helps in the selection of developing neurons [149].

The incorporation of platelet factors into the field of neuroscience highlights promising therapeutic possibilities. In addition to BDNF, other platelet-derived elements, such as Platelet Factor 4 (PF4), play an essential role in neurobiology, in particular by regulating hippocampal neurogenesis and cognitive functions [150]. Using their neurotrophic factors and cytokines, platelets are able to influence brain function and pathology by regulating the integrity and function of the blood-brain barrier (BBB) [151]. The complex interaction between brain and blood suggests that platelets play a role not only in hemostasis and immune responses, but also in brain plasticity and repair mechanisms. The potential use of platelet lysates and platelet-derived extracellular vesicles (EVs) is also highlighted by recent studies [90,152]. Platelet lysates, which contain large quantities of growth factors and cytokines, have therapeutic potential for tissue restoration and wound healing [90]. In addition, platelet EVs, rich in proteins, RNA and lipids, have proven their ability to regulate immune responses and promote neuroprotection [152]. It is possible to use these platelet-derived products to enhance neurogenesis and neuroprotection in situations such as stroke and traumatic brain injury, representing an innovative approach to the treatment of neurodegenerative diseases.

### 8.2 Future directions and research gaps

These close links between neuronal and platelet physiology suggest opportunities to use platelets, readily available in circulation, as biomarkers of cognitive health or even to modulate platelet activity to use them as biological vectors for neuroactive factors.

Future studies should focus on the following.

- Explaining the specific processes by which platelet-derived elements impact brain function and disease.
- Adapting to species and experimental conditions to standardize methods for measuring BDNF and other platelet-derived factors.
- Integrate platelet lysates and EVs into clinical studies for neurological and psychiatric conditions.
- Analyze the function of platelets in BBB regulation and its implications for drug delivery and neuronal protection.

By exploring these areas, we will be able to deepen our understanding of the complex interaction between platelets and brain health, enabling the development of innovative treatments for neurological conditions.

## 9 Conclusion

This review highlights the diverse role of platelets in neurological disorders, focusing on their role as a storehouse of BDNF and other neurotrophic factors. Platelet-derived BDNF has a significant impact on neuroplasticity, neuroprotection and cognitive function, with implications for various neurological and psychiatric conditions.

## CRediT authorship contribution statement

**Nezha Bouhaddou:** Writing – original draft, Visualization, Validation, Conceptualization. **Meryem Mabrouk:** Writing – review & editing, Writing – original draft, Visualization, Loubna Khalki, Writing – review & editing, Writing – original draft, Validation, Supervision. **Farah Atifi:** Writing – review & editing, Visualization. **Abdelhakim Bouyahya:** Writing – review & editing, Visualization, Validation. **Younes Zaid:** Writing – review & editing, Writing – original draft, Visualization, Validation, Supervision, Nabil Zaid, Writing – review & editing, Writing – original draft, Validation, Khang Wen Goh, Writing – review & editing, Writing – original draft, Resources.

## Funding

This research received no external funding

## Declaration of competing interest

The authors declare that they have no known competing financial interests or personal relationships that could have appeared to influence the work reported in this paper.
